# The historical and contemporary aspects of cognitive behavioral therapy for insomnia in recent 30 years: a bibliometric study

**DOI:** 10.3389/fpsyt.2026.1878379

**Published:** 2026-08-05

**Authors:** Houjian He, Maorong Hu, Shansi Li, Faguo Yue

**Affiliations:** 1 Sleep and Psychology Center, Bishan Hospital of Chongqing Medical University, Chongqing, China; 2 Department of Psychiatry, The First Affiliated Hospital of Nanchang University, Nanchang, China

**Keywords:** behavioral therapy, bibliometric analysis, CBT-I, cognitive therapy, insomnia, intellectual base, research front, research hotspot

## Abstract

**Objective:**

Cognitive Behavioral Therapy for Insomnia (CBT-I) has become the leading intervention for insomnia over the past two decades, with well-established efficacy. Yet, no systematic approach exists to rapidly capture its intellectual foundations, research frontiers and research hotspots. This study addresses this gap through bibliometric analysis.

**Method:**

Using a comprehensive set of synonyms and related terms for CBT-I as search keywords, a methodical literature search was carried out. The search was restricted to review papers and articles published in peer-reviewed journals between the years 1996 and 2025. This resulted in 6996 records from Scopus and 4595 records from the Web of Science(WoS) Core Collection. CiteSpace was applied to analyze cited references and terms. VOSviewer was applied to analyze the co-authorship in authors, organizations and countries/regions.

**Results:**

(a)Eight primary knowledge bases were identified, encompassing management guidelines and reference books on insomnia; face-to-face CBT-I interventions for individuals with insomnia; self-help or digital CBT-I interventions for insomnia; CBT-I applications in mental disorders comorbid insomnia; CBT-I interventions for insomnia comorbid with medical conditions; the overview of information about insomnia; sleep assessment tools; and other related topics. (b)Three emerging research frontiers in the CBT-I domain over the past 5 years include: digital delivery of CBT-I interventions, the practice of CBT-I in sleep apnea, and CBT-I among cancer populations. (c)Morin CM is the leading researcher in the field; University of California has emerged as the leading institution in CBT-I research; the United States collectively produces the highest volume of CBT-I related research globally. (d)Current research hotspots in CBT-I include: adding CBT-I applications to obstructive sleep apnea (OSA), the practice of CBT-I during the COVID-19 epidemic, population-specific modifications of CBT-I for at-risk teenagers, new studies on CBT-I’s digital delivery methods, assessing CBT-I’s effectiveness and adaptation in comorbid psychiatric diseases (especially anxiety-related conditions).

**Conclusion:**

This study delineates the intellectual base and evolving trends of CBTI research. The results help beginners quickly grasp the field’s development, improve practice accuracy and consistency, and provide researchers with a comprehensive overview to inform future innovations.

## Introduction

With the widespread dissemination of cognitive behavioral therapy (CBT), a specialized application—Cognitive Behavioral Therapy for Insomnia (CBT-I)—was developed. Empirical support for CBT-I has accumulated steadily since the 1960s (1), and it is now recognized by the American College of Physicians (ACP) as the first-line treatment for insomnia (2). The standard CBT-I model includes cognitive interventions targeting maladaptive sleep beliefs, behavioral strategies such as sleep restriction and stimulus control, and educational components such as sleep hygiene (3). To meet diverse patient needs, CBT-I has been adapted into multiple formats, including individual and group therapy, telephone- and web-based delivery (4), and self-help approaches (5).

Over decades of research, a substantial body of literature has accumulated, and numerous systematic reviews have synthesized CBT-I evidence from various perspectives. However, most reviews focus on narrow topics and draw on a limited set of studies, resulting in high specificity but limited generalizability. This fragmentation prevents the integration of different research directions into a comprehensive overview. To address this gap, the present study employs bibliometric analysis to systematically incorporate the full body of CBT-I literature, providing a holistic mapping of the field and an accessible entry point for newcomers.

Both VOSviewer and CiteSpace are widely used bibliometric and visualization tools; however, VOSviewer excels in analyzing static co-occurrence relationships, whereas CiteSpace is particularly oriented toward dynamic time-series analysis and the detection of emerging research frontiers (6). In light of their respective strengths, this study employs CiteSpace to analyze cited references and terms in the CBT-I domain to identify its intellectual foundations, research fronts, and thematic hotspots. VOSviewer is utilized to examine co-authorship networks within the CBT-I field, aiming to reveal collaborative research patterns and institutional cooperation structures.

To avoid repetitive and meaningless research, this study conducted a thorough review of existing literature and identified only one comparable study, titled “The 100 Most Cited Papers in Insomnia: Bibliometric Analysis” (7). Nevertheless, there are notable distinctions between the two studies. First, their research focuses differ: this study centers on CBT-I, whereas Wan et al. investigates insomnia more broadly, encompassing a larger scope. Second, the research objectives are distinct. This study aims to explore the knowledge base and emerging research frontiers in the field of CBT-I, with a particular emphasis on cited references. In contrast, Wan et al. seeks to provide a comprehensive analysis of the general characteristics of insomnia-related research. Therefore, this study retains independent academic value and contributes uniquely to the existing body of knowledge.

## Methods

### Bibliometric data collection

First, this study selected the Web of Science (WoS) Core Collection database (including SCIE, SSCI, and ESCI) and Scopus as primary data sources. The selection of these databases was based on their complementary strengths: WoS applies more rigorous inclusion criteria and places greater emphasis on assessing scholarly influence and academic impact, whereas Scopus provides broader coverage of the literature and employs a wider range of evaluation metrics (8). Second, only articles and reviews were included in the analysis. This decision was driven by the study’s focus on citation networks, as other document types—such as meeting abstracts, editorials, letters, and conference proceedings—typically lack comprehensive reference lists. Third, two researchers independently screened the retrieved records and cross-verified the results to ensure methodological rigor and consistency.

The search strategy implemented on June 14, 2026, was as follows:

In the WoS (Science Citation Index Expanded [SCI-EXPANDED], 1990–present; Social Sciences Citation Index [SSCI], 1990–present; Emerging Sources Citation Index [ESCI], 2015–present), the search query was: (TS = (“Cognitive Therapy” and “Insomnia”) OR TS = (“Behavio* Therapy” and “Insomnia”) OR TS = (“CBT” and “Insomnia”) OR TS = “Stimulus Control Therapy” OR TS = “Sleep Restriction Therapy” OR TS = “CBT-I”) AND DOCUMENT TYPES: (Article OR Review). Timespan: 1996-2025. After removing duplicates and irrelevant records, 4595 valid articles were retained. Data were exported in plain text format, including full records and cited references.

In Scopus, the search strategy was: (TITLE-ABS-KEY(“Cognitive Therapy” AND “Insomnia”) OR TITLE-ABS-KEY(“stimulus control therapy”) OR TITLE-ABS-KEY(“sleep restriction therapy”) OR TITLE-ABS-KEY(“Behavio* Therapy” AND “Insomnia”) OR TITLE-ABS-KEY(“CBT-I”) OR TITLE-ABS-KEY(“CBT” AND “insomnia”)) AND PUBYEAR >1995 AND PUBYEAR < 2026 AND (LIMIT-TO(DOCTYPE, “ar”) OR LIMIT-TO(DOCTYPE, “re”)). Following screening, 6996 eligible articles were included. These records were saved in CSV format with complete metadata.

### Data analysis

The analysis of cited references and terms was conducted using CiteSpace (version 6.4.R1), while the co-authorship in authors, organizations and countries/regions was performed using VOSviewer (version 1.6.20). To preserve data diversity, no language restrictions were applied to the selected dataset in this study. Given that the Scopus database contains citation data in multiple languages—particularly in non-English articles—conducting comprehensive citation analysis on such data presents significant challenges. In contrast, all records in the WoS are in English, thereby avoiding potential linguistic inconsistencies. Therefore, only data sourced from the WoS were utilized in cited reference analysis.

CiteSpace’s parameter configurations were established as follows: (a) A one-year time-slicing interval was used in this study to ensure complete annual coverage of the literature and to mitigate potential bias from inter-annual variations in publication volume. (b) In accordance with the software developer’s recommendation, the number of nodes retained for analysis was limited to approximately 1, 000. This was sufficient to maintain analytical robustness while avoiding visual clutter and information redundancy (caused by excessive nodes) and the loss of crucial scholarly signals and the ensuing analytical distortion (caused by inadequate nodes). As a result, term analysis used a k-value of 20 and cited reference analysis used a k-value of 10, both empirically calibrated to keep node counts close to the target threshold. (c) No nodes were pruned or filtered, preserving the raw network structure’s integrity and minimizing methodological artifacts in the visualization and interpretation of results. In short, time slicing spanned from January 1996 to December 2025, with one-year intervals; the selection criterion employed was the g-index, with a k-value of 10 or 20; the analysis focused on two node types: cited references and terms; no pruning methods were applied; and all other parameters were retained at their default settings.

For VOSviewer, the co-authorship network analysis was configured with minimum thresholds of ≥20 publications for countries, ≥20 for institutions, and ≥20 for authors.

## Results

### Introduction of data

**Figure 1:**
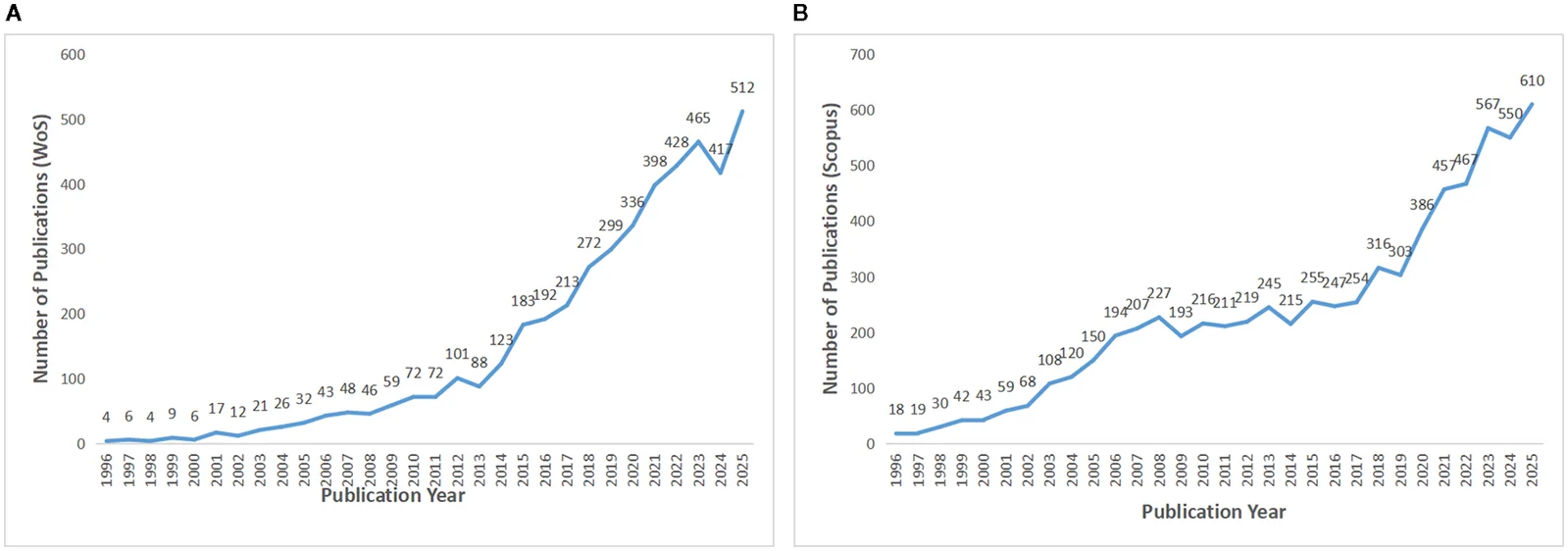
Annual publication trends of CBT-I related research from 1996 to 2025. **(A)** Annual publication trends in WoS. **(B)** Annual publication trends in Scopus.

An examination of publication trends across the WoS and Scopus databases reveals the pattern illustrated in **Figure 1**. Overall, a consistent year-on-year growth is evident. The knowledge evolution and development in the field of CBTI can be simply divided into three stages: the early stage of CBT-I research (1996-2005), the mid-term period (2006-2015) and the recent period (from 2016-2025). The analysis of the development process of CBT-I in the following text will all adopt this time stage division standard.

**Figure 2:**
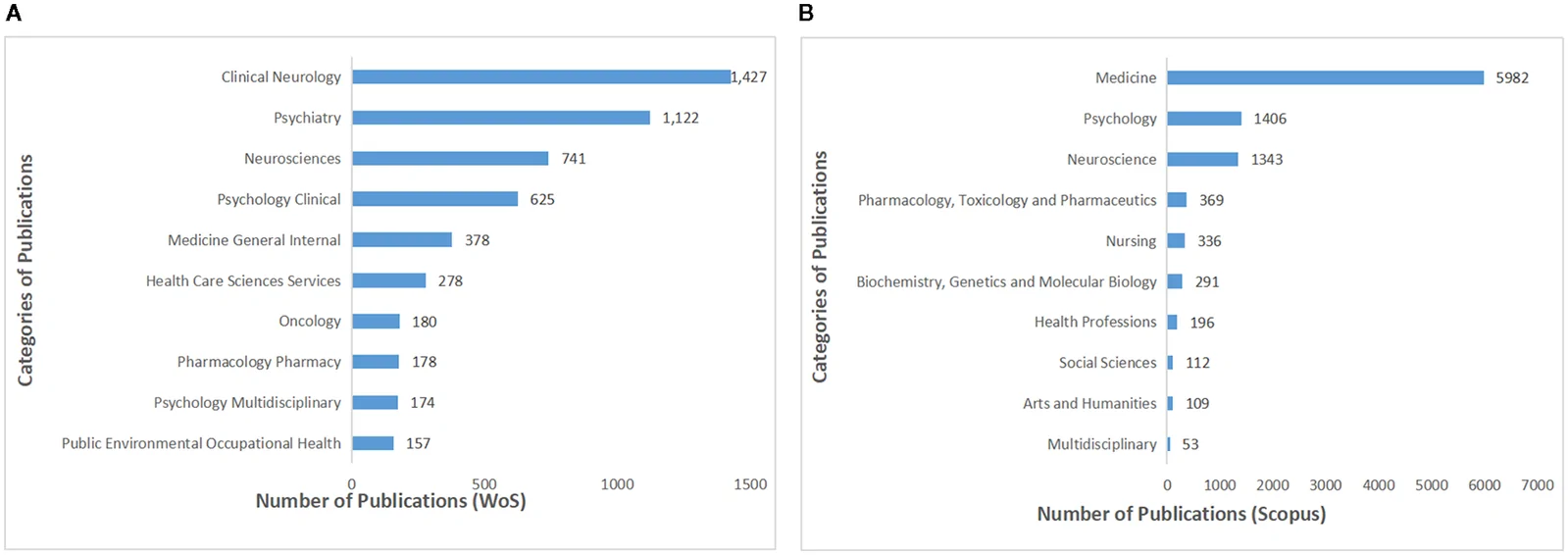
Top 10 in category distribution of CBT-I related research. **(A)** Category distribution in WoS. **(B)** Category distribution in Scopus.

Based on the subject classification systems of each database, **Figure 2** displays the top ten subject categories. In the WoS database, “Clinical Neurology” is the most prominent discipline, whereas in Scopus, “Medicine” accounts for the highest number of publications.

### Analysis of cited references

**Figure 3:**
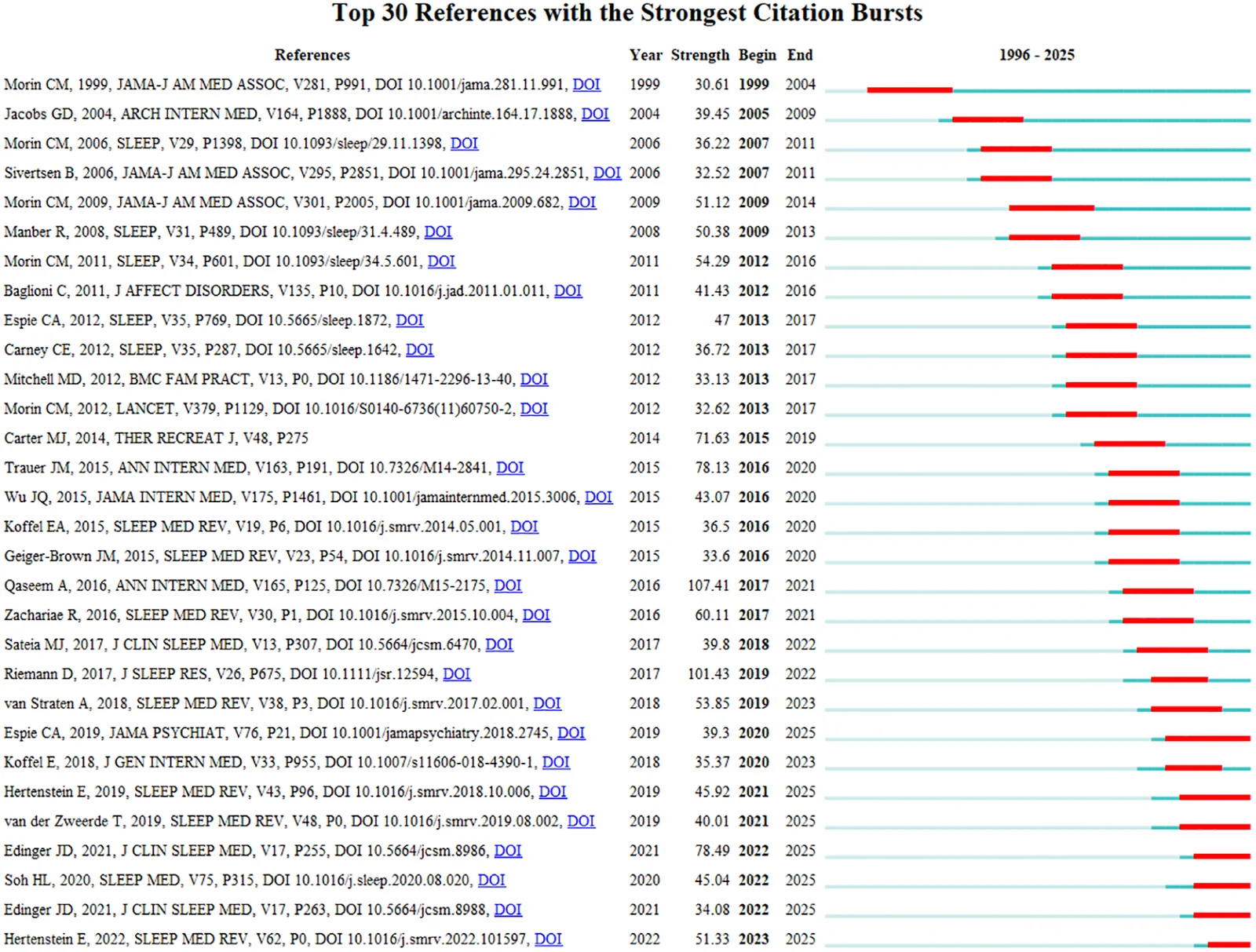
Top 30 references with the strongest citation bursts in CBT-I based on WoS.

Co-citation refers to “the frequency with which two items of earlier literature are cited together by later literature” (9). The intellectual base of a field is constituted by co-cited articles, while the research front consists of articles citing this intellectual base. Reference co-citation analysis thus reveals the intellectual foundations and emerging directions of a domain. **Table 1** listed the top 50 most cited articles on CBT-I, **Figure 3** listed the top 30 articles with strongest citation bursts on CBT-I. The article with the highest citation count and strongest citation bursts is titled “management of chronic insomnia disorder in adults: a clinical practice guideline from the American college of physicians” (3).

**Table 1:** the top 50 most cited references in CBTI.

Total citations	First Author	Published year	Title	DOI
299	Qaseem A	2016	Management of chronic insomnia disorder in adults: a clinical practice guideline from the American College of Physicians (3).	10.7326/M15-2175
282	Riemann D	2017	European guideline for the diagnosis and treatment of insomnia (10).	10.1111/jsr.12594
241	Edinger JD	2021	Behavioral and psychological treatments for chronic insomnia disorder in adults: an American Academy of Sleep Medicine clinical practice guideline (11).	10.5664/jcsm.8986
204	van Straten A	2018	Cognitive and behavioral therapies in the treatment of insomnia: a meta-analysis (12).	10.1016/j.smrv.2017.02.001
198	Trauer JM	2015	Cognitive behavioral therapy for chronic insomnia: a systematic review and meta-analysis (13).	10.7326/M14-2841
181	Zachariae R	2016	Efficacy of internet-delivered cognitive-behavioral therapy for insomnia: a systematic review and meta-analysis of randomized controlled trials (14).	10.1016/j.smrv.2015.10.004
180	Carter M	2014	Diagnostic and statistical manual of mental disorders, 5th ed (15).	
159	Soh HL	2020	Efficacy of digital cognitive behavioural therapy for insomnia: a meta-analysis of randomised controlled trials (16).	10.1016/j.sleep.2020.08.020
149	Hertenstein E	2019	Insomnia as a predictor of mental disorders: a systematic review and meta-analysis (17).	10.1016/j.smrv.2018.10.006
149	Riemann D	2023	The European insomnia guideline: an update on the diagnosis and treatment of insomnia 2023 (18).	10.1111/jsr.14035
148	Espie CA	2019	Effect of digital cognitive behavioral therapy for insomnia on health, psychological well-being, and sleep-related quality of life: a randomized clinical trial (19).	10.1001/jamapsychiatry.2018.2745
135	Sateia MJ	2017	Clinical practice guideline for the pharmacologic treatment of chronic insomnia in adults: an American Academy of Sleep Medicine clinical practice guideline (20).	10.5664/jcsm.6470
121	Hertenstein E	2022	Cognitive behavioral therapy for insomnia in patients with mental disorders and comorbid insomnia: a systematic review and meta-analysis (21).	10.1016/j.smrv.2022.101597
110	Wu JQ	2015	Cognitive behavioral therapy for insomnia comorbid with psychiatric and medical conditions: a meta-analysis (22).	10.1089/acm.2014.0251
106	van der Zweerde T	2019	Cognitive behavioral therapy for insomnia: a meta-analysis of long-term effects in controlled studies (23).	10.1016/j.smrv.2019.08.002
104	Koffel EA	2015	A meta-analysis of group cognitive behavioral therapy for insomnia (24).	10.1016/j.smrv.2014.05.001
102	Morin CM	2011	The insomnia severity index: psychometric indicators to detect insomnia cases and evaluate treatment response (25).	10.1093/sleep/34.5.601
98	Edinger JD	2021	Behavioral and psychological treatments for chronic insomnia disorder in adults: an American Academy of Sleep Medicine systematic review, meta-analysis, and GRADE assessment (26).	10.5664/jcsm.8988
96	Koffel E	2018	Increasing access to and utilization of cognitive behavioral therapy for insomnia (CBT-I): a narrative review (27).	10.1007/s11606-018-4390-1
92	Espie CA	2012	A randomized, placebo-controlled trial of online cognitive behavioral therapy for chronic insomnia disorder delivered via an automated media-rich web application (28).	10.5665/sleep.1872
91	Ritterband LM	2017	Effect of a web-based cognitive behavior therapy for insomnia intervention with 1-year follow-up: a randomized clinical trial (29).	10.1001/jamapsychiatry.2016.3249
91	Morin CM	2009	Cognitive behavioral therapy, singly and combined with medication, for persistent insomnia (30).	10.1001/jama.2009.682
88	Wilson S	2019	British Association for Psychopharmacology consensus statement on evidence-based treatment of insomnia, parasomnias and circadian rhythm disorders: an update (31).	10.1177/0269881119855343
86	Geiger-Brown JM	2015	Cognitive behavioral therapy in persons with comorbid insomnia: a meta-analysis (32).	10.1016/j.smrv.2014.11.007
86	Christensen H	2016	Effectiveness of an online insomnia program (SHUTi) for prevention of depressive episodes (the Good Night Study):a randomised controlled trial (33).	10.1016/S2215-0366(15)00536-2
85	Manber R	2008	Cognitive behavioral therapy for insomnia enhances depression outcome in patients with comorbid major depressive disorder and insomnia (34).	10.1093/sleep/31.4.489
83	Freeman D	2017	The effects of improving sleep on mental health (OASIS):a randomised controlled trial with mediation analysis (35).	10.1016/S2215-0366(17)30328-0
83	Seyffert M	2016	Internet-delivered cognitive behavioral therapy to treat insomnia: a systematic review and meta-analysis (36).	10.1371/journal.pone.0149139
83	Morin CM	2022	Epidemiology of insomnia: prevalence, course, risk factors, and public health burden (37).	10.1016/j.jsmc.2022.03.003
79	Cunningham JEA	2018	Cognitive behavioural therapy for insomnia (CBT-I) to treat depression: a systematic review (38).	10.1016/j.jpsychores.2017.12.012
79	Johnson JA	2016	A systematic review and meta-analysis of randomized controlled trials of cognitive behavior therapy for insomnia (CBT-I) in cancer survivors (39).	10.1016/j.smrv.2015.07.001
78	Baglioni C	2011	Insomnia as a predictor of depression: a meta-analytic evaluation of longitudinal epidemiological studies (40).	10.1016/j.jad.2011.01.011
75	Ho FYY	2015	Self-help cognitive-behavioral therapy for insomnia: a meta-analysis of randomized controlled trials (5).	10.1016/j.smrv.2014.06.010
75	Baglioni C	2020	The European academy for cognitive behavioural therapy for insomnia: an initiative of the european insomnia network to promote implementation and dissemination of treatment (41).	10.1111/jsr.12967
73	Hasan F	2022	Comparative efficacy of digital cognitive behavioral therapy for insomnia: a systematic review and network meta-analysis (42).	10.1016/j.smrv.2021.101567
72	Carney CE	2012	The consensus sleep diary: standardizing prospective sleep self-monitoring (43).	10.5665/sleep.1642
67	Selvanathan J	2021	Cognitive behavioral therapy for insomnia in patients with chronic pain–a systematic review and meta-analysis of randomized controlled trials (44).	10.1016/j.smrv.2021.101460
65	Garland SN	2014	Mindfulness-based stress reduction compared with cognitive behavioral therapy for the treatment of insomnia comorbid with cancer: a randomized, partially blinded, noninferiority trial (45).	10.1200/JCO.2012.47.7265
65	Mitchell MD	2012	Comparative effectiveness of cognitive behavioral therapy for insomnia: a systematic review (46).	10.1186/1471-2296-13-40
64	Morin CM	2012	Chronic insomnia (47).	10.1016/S0140-6736(11)60750-2
63	Talbot LS	2014	Cognitive behavioral therapy for insomnia in posttraumatic stress disorder: a randomized controlled trial (48).	10.5665/sleep.3408
63	Jacobs GD	2004	Cognitive behavior therapy and pharmacotherapy for insomnia: a randomized controlled trial and direct comparison (49).	10.1001/archinte.164.17.1888
61	Perlis ML	2022	Insomnia (50).	10.1016/S0140-6736(22)00879-0
61	Vedaa O	2020	Effects of digital cognitive behavioural therapy for insomnia on insomnia severity: a large-scale randomised controlled trial (51).	10.1016/S2589-7500(20)30135-7
60	Freeman D	2020	Sleep disturbance and psychiatric disorders (52).	10.1016/S2215-0366(20)30136-X
59	Taylor DJ	2014	Cognitive and behavioural therapy for insomnia (CBT-I) in psychiatric populations: a systematic review (53).	10.3109/09540261.2014.902808
59	Morin CM	2006	Psychological and behavioral treatment of insomnia: update of the recent evidence (1998–2004) (54).	10.1093/sleep/29.11.1398
59	Buysse DJ	2011	Efficacy of brief behavioral treatment for chronic insomnia in older adults (55).	10.1001/archinternmed.2010.535
58	American Academy of Sleep Medicine	2014	International classification of sleep disorders, 3rd ed. Darien: American Academy of Sleep Medicine (56).	
57	De Crescenzo F	2022	Comparative effects of pharmacological interventions for the acute and long-term management of insomnia disorder in adults: a systematic review and network meta-analysis (57).	10.1016/S0140-6736(22)00878-9
57	Zachariae R	2018	Internet-delivered cognitive-behavioral therapy for insomnia in breast cancer survivors: a randomized controlled trial (58).	10.1093/jnci/djx293

Based on the topics addressed, the 50 cited articles can be classified into 8 categories: (a) management guidelines and reference books on insomnia (3, 10, 11, 15, 18, 20, 31, 41, 56); (b) face-to-face CBT-I in individuals with insomnia disorder (12, 13, 23, 24, 26, 30, 46, 49, 53, 54); (c) self-help or digital CBT-I interventions for insomnia (5, 14, 16, 28, 29, 36, 42, 51); (d) CBT-I interventions in mental disorders comorbid insomnia (19, 21, 22, 32–35, 38, 48, 53); (e) CBT-I interventions in insomnia comorbid with medical disorders, such as cancer and chronic pain (22, 32, 39, 44, 45, 55, 58); (f)the overview of information about insomnia (17, 37, 40, 47, 50, 52); (g)sleep assessment tools (25, 43); and (h) other relevant topics (27, 57).

To explore the evolutionary trajectory of highly cited literature in the field of CBT-I and to identify seminal works that have played pivotal roles at different developmental stages, this study conducted a burstness analysis based on co-citation networks. According to the findings, the publications exhibiting the high citation burstsduring the early CBT-I research period (from 1996 to 2005) primarily focused on comparative evaluations of the efficacy of CBT-I versus pharmacological treatments for insomnia, as well as investigations into the effects of combining CBT-I and medication therapies (49, 59). These initial studies established CBT-I’s clinical efficacy and served as crucial points of reference for subsequent research aimed at expanding its applications, making them essential sources in the field.

During the intermediate phase (2006-2015), key assessment instruments for measuring insomnia intervention outcomes—such as the Insomnia Severity Index (ISI) and sleep diaries—were widely adopted in early CBT-I studies and demonstrated significant citation bursts, highlighting their methodological significance (25, 43). Simultaneously, researchers began conducting systematic reviews of initial randomized controlled trials to consolidate evidence-based support for the clinical implementation of CBT-I (46). As a result, medical institutions globally developed a series of clinical practice guidelines for insomnia management. These review articles and clinical guidelines subsequently served as fundamental resources for follow-up research. Concurrently, with advances in internet technology and its widespread adoption, growing attention has been directed toward integrating digital platforms into CBT-I delivery, leading to the emergence of digital and self-guided CBT-I programs (28). This innovation has substantially expanded the accessibility and reach of CBT-I, rendering related literature particularly prominent in citation burst patterns.

In the past 10 years, the most frequently cited works with the strongest citation bursts in CBT-I research fall into two primary categories: systematic reviews on digital CBT-I and updated clinical practice guidelines. These two types of publications often engage in reciprocal citation—evidence derived from digital CBT-I studies informs the revision of clinical guidelines, while updated guidelines promote further clinical application and evaluation of digital CBT-I—thereby forming a mutually reinforcing cycle that drives continuous progress in the field.

### Cluster analysis of cited references

**Figure 4:**
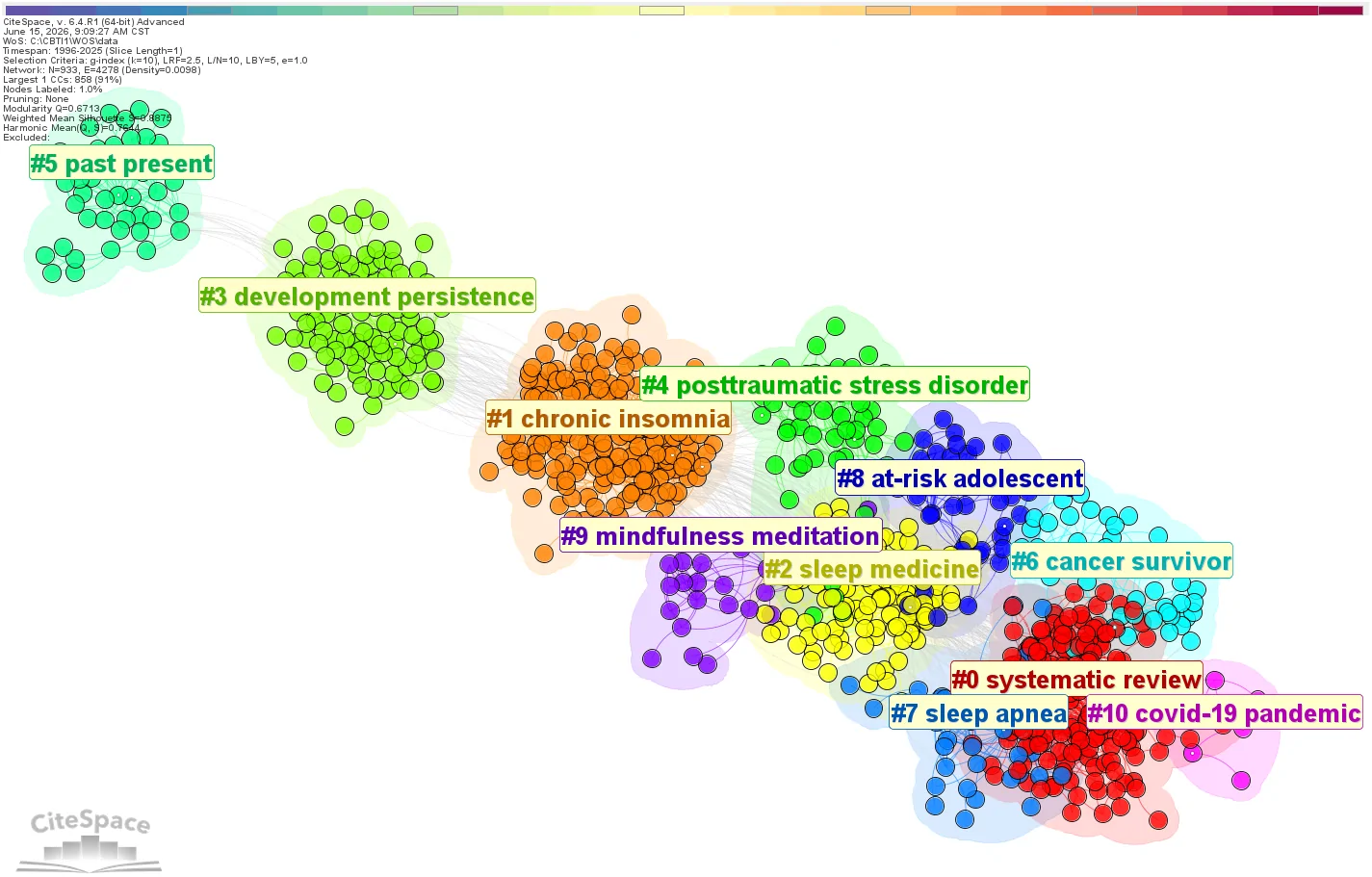
Cluster of cited references in CBTI.

**Figure 5:**
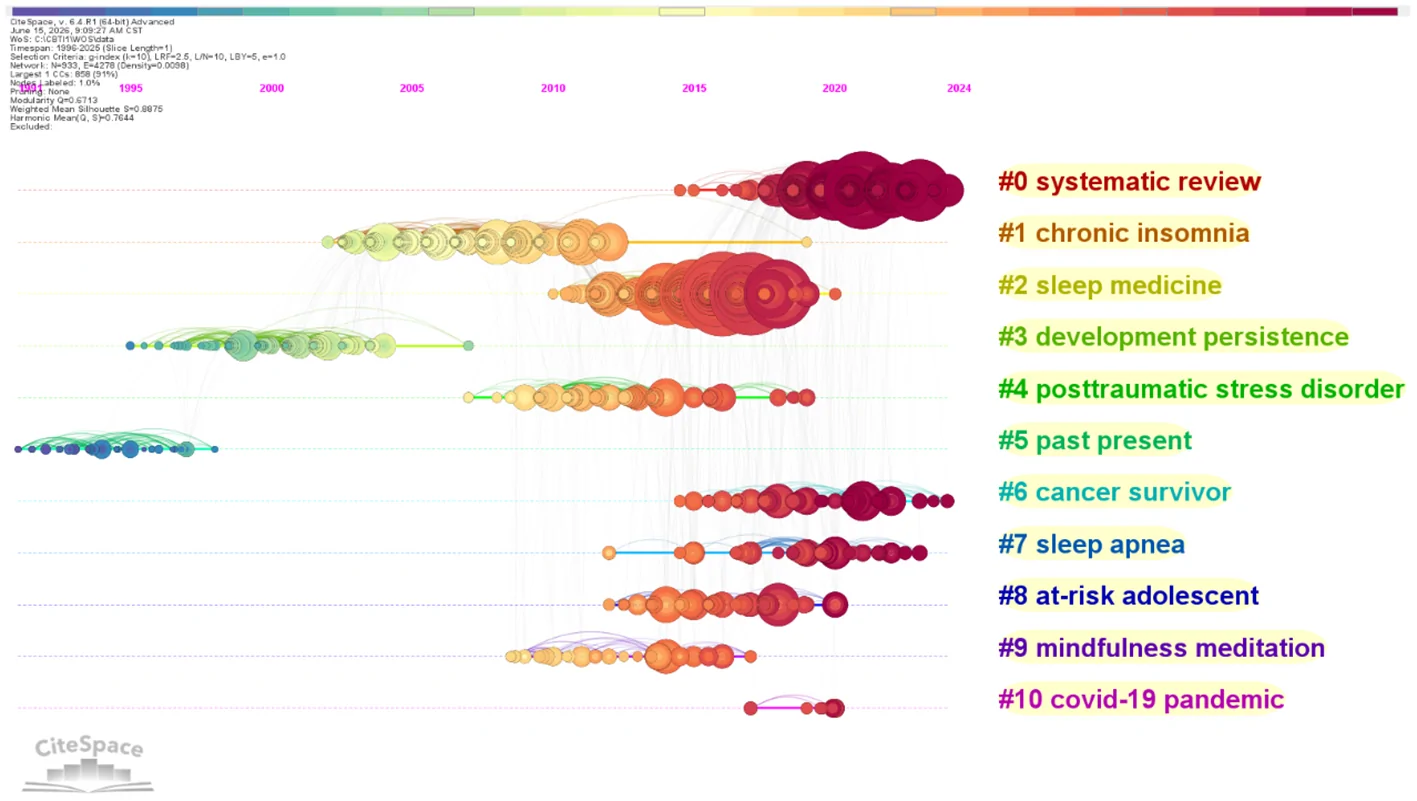
Timeline of cluster of cited references in CBTI.

Based on the analysis of cited literature, cluster analysis was conducted. During the clustering process, the log-likelihood ratio (LLR) algorithm extracts multiple noun phrases from the keywords of citing literature, computes their corresponding log-likelihood ratio values, and subsequently presents the top 5 noun phrases with the highest log-likelihood ratio scores, enabling researchers to select appropriate clustering labels. **Figure 4** and **Figure 5** presented 11 co-citation clusters, while **Table 2** provided five alternative noun phrases for each corresponding cluster label.

**Table 2:** Summury of cluster in cited references in CBTI.

Cluster ID	Size	Silhouette	Mean (year)	Top 5 terms (log-likelihood ratio)
0	181	0.835	2020	systematic review (3458.19); digital cognitive behavioral therapy (2940.09); dsm-5 insomnia disorder (1713.98); university student (1594.47); digital sleep intervention (1321.66)
1	179	0.913	2006	chronic insomnia (1668.16); sleep disorder (1379.91); sleep restriction therapy (1349.49); cognitive-behavior therapy (1348.45); systematic review (1151.77)
2	133	0.805	2014	sleep medicine (1275.51); long-term effect (1264.97); wait-list controlled trial (1176.7); cancer survivor (1137.83); internet-delivered cognitive-behavioral therapy (1114.39)
3	102	0.942	2000	development persistence (427.47); conceptual issue (427.47); measuring outcome (416.76); cognitive behaviour therapy (373.92); benzodiazepine discontinuation (363.22)
4	60	0.901	2012	posttraumatic stress disorder (2231.7); post-traumatic stress disorder (1698.71); posttraumatic stress (1300.83); sleep disturbance (931.99); substance use disorder (852.07)
5	46	1	1994	past present (119.28); behavioral insomnia therapy (119.28); pharmacological therapy (85.06); critical issue (68); combined effect (68)
6	46	0.894	2019	cancer survivor (5921.97); cancer patient (1376.22); chronic pain (997.65); exploratory analysis (944.83); breast cancer (854.55)
7	38	0.938	2019	sleep apnea (4695.47); co-morbid insomnia (2423.38); sleep apnoea (2193.74); obstructive sleep apnea (1479.19); comorbid insomnia (1470.09)
8	37	0.895	2016	at-risk adolescent (1112.6); mental health service (888.11); treatment research (605.26); sense study (589.1); assessing sleep (573.52)
9	26	0.928	2012	mindfulness meditation (1367.64); cancer patient (661.15); bests practice (434.09); insomnia outcome (425.38); mindfulness-based cancer recovery (407.98)
10	7	0.992	2019	covid-19 pandemic (303.85); brazilian physiotherapist (168.28); health care worker (154.23); single centers experience (154.23); covid-19 epidemic (154.23)

The following selection strategy was used by researchers to select the most representative label for each cluster: (a) the noun phrase with the highest LLR score is chosen as the cluster label; (b) if multiple noun phrases convey similar meanings, their meanings are synthesized into a single label; (c) the common meaning is retained as the label if the results from the two methods (strategy a and b) are consistent or highly similar. Both are retained as co-labels in the event that their meanings significantly diverge. (d) methodological descriptors (e.g., systematic review) were systematically excluded from the cluster analysis.

Based on the average publication year and timeline of the cited literature within each cluster, 3 clusters (Cluster #0, #6 and #7) exhibit mean values falling within the past 5 years (2021-2025), indicating their relevance to recent research developments. According to the clustering label generation criteria, these clusters can be interpreted as representing key research frontiers in the field over the last 5 years. Studies that focus on digital CBT-I model is characteristic of Cluster #0. Cluster #6 focuses on cancer patients who use CBTI to treat insomnia. Sleep apnea-related insomnia is the focus of Cluster #7.

## The analysis of co-authorship

The analysis of co-authorship among authors, organizations, and countries/regions can help identify key contributors and collaborative networks within the current research landscape, offering valuable insights and potential collaboration opportunities for scholars encountering research challenges or those newly entering the field.

### Co-authorship among authors

**Figure 6:**
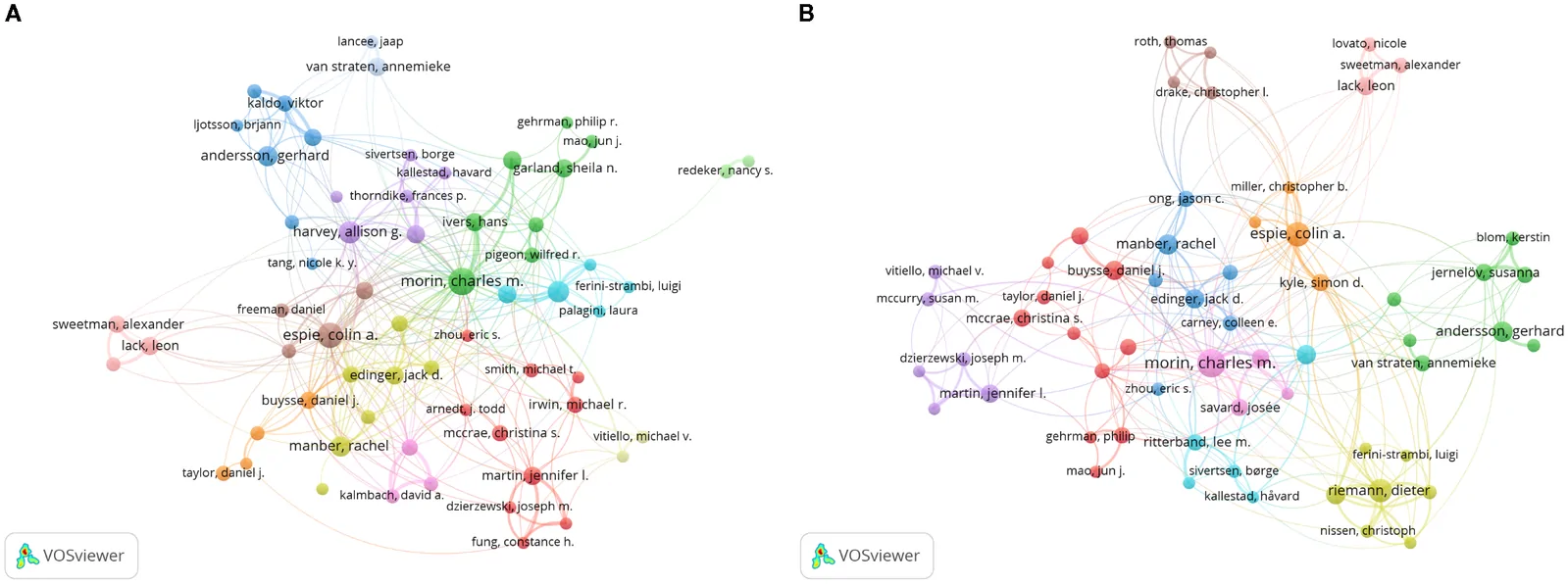
Author networks of research outputs in CBT-I studies(1996-2025): **(A)** Co-authorship network analysis using WoS. **(B)** Co-authorship network analysis using Scopus.

An analysis of author co-authorship based on two databases yielded consistent findings: Morin CM, Espie CA, and Riemann D are the top three most prolific scholars in the CBT-I field. The publication output of other authors and their collaborative networks are presented in **Table 3** and **Figure 6**.

**Table 3:** Top 10 Authors with the highest number of publications in CBT-I related research.

WoS		Scopus	
Author	Articles	Author	Articles
Morin, Charles M. (Laval University, Canada)	100	Morin, Charles M. (Laval University, Canada)	112
Espie, Colin A. (University of Oxford, UK)	87	Espie, Colin A. (University of Oxford, UK)	82
Harvey, Allison G. (University of Virginia, USA)	67	Riemann, Dieter (University of Freiburg, Germany)	74
Riemann, Dieter (University of Freiburg, Germany)	61	Andersson, Gerhard (Karolinska Institutet, Sweden)	57
Andersson, Gerhard (Karolinska Institutet, Sweden)	56	Manber, Rachel (Stanford University, USA)	54
Manber, Rachel (Stanford University, USA)	55	Harvey, Allison G (University of Virginia, USA)	53
Spiegelhalder, Kai (University of Freiburg, Germany)	47	Edinger, Jack D (National Jewish Health, USA)	52
Edinger, Jack D. (National Jewish Health, USA)	46	Buysse, Daniel J (University of Pittsburgh, USA)	51
Ivers, Hans (Laval University, Canada)	45	Spiegelhalder, Kai (University of Freiburg, Germany)	50
Ritterband, Lee M. (University of Virginia, USA)	45	Lack, Leon C. (Flinders University, Australia)	47

Morin CM currently holds a position as a full-time professor in the Department of Psychology at Laval University, Canada, and serves as the Canada Research Chair in Sleep Disorders. Previously, he was an associate professor in the Department of Psychiatry at Virginia Commonwealth University in the United States. Dr. Morin’s team has elucidated the mechanisms underlying insomnia and developed two assessment tools: ISI and dysfunctional beliefs and attitudes about sleep(DBAS-16) (60). He has also authored several reference books for clinicians and self-help guides on insomnia, such as “Conquering the Enemies of Sleep.” Additionally, Dr. Morin has collaborated on subsequent validations and meta-analyses about SHUTi (Sleep Healthy Using The Internet), to provide accessible treatment options for insomnia patients. His research aims to develop therapeutic approaches to assist individuals with insomnia. From the perspective of author collaboration networks, Morin’s closest collaborator is Ivers Hans, with whom he has co-authored over 20 articles since their first joint publication in 2007. Together, they validated a brief version of the DBAS-16. Other key collaborators of Morin include Savard Josée, another colleague, and Ritterband Lee M from the University of Virginia, USA. Additionally, Morin has collaborated with Espie Colin A from the University of Oxford, UK—ranked second in CBT-I publications—and Riemann Dieter from the University of Freiburg, Germany—ranked third.

### Co-authorship among organizations

**Figure 7:**
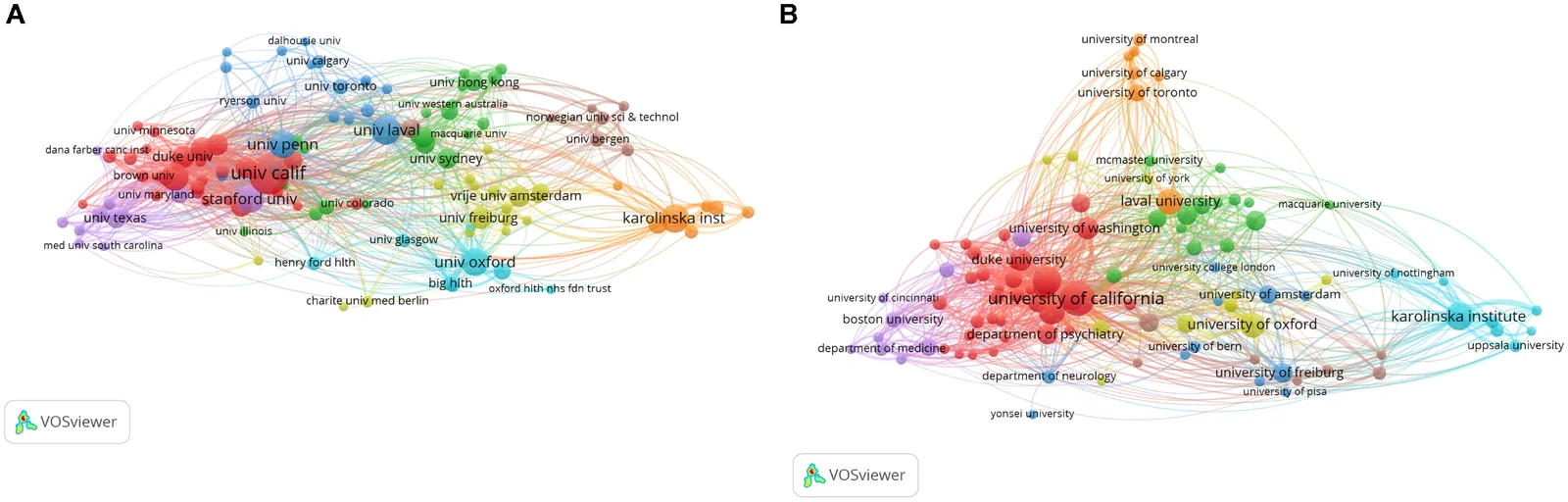
Institutional contributions of research outputs in CBT-I studies(1996-2025): **(A)** Inter-institutional collaboration network derived from WoS. **(B)** Inter-institutional collaboration network derived from Scopus.

The two databases’ analyses of organizational co-authorship reveal some consistency in regards to the top three publishing institutions. The University of California comes in first in both databases, followed by Karolinska Institutet in third. However, Laval University comes in second in WoS, while Harvard University holds that position in Scopus. The publication counts and collaborative networks of other organizations are detailed in **Table 4** and **Figure 7**.

**Table 4:** Top 10 organizations with the highest number of publications in CBT-I related research.

WoS		Scopus	
Organizations	Articles	Organizations	Articles
University of California(USA)	316	University of California(USA)	259
Laval University(Canada)	172	Harvard University(USA)	179
Karolinska Institutet(Sweden)	161	Karolinska Institutet(Sweden)	167
Stanford University(USA)	158	University of Pennsylvania(USA)	159
University of Pennsylvania (USA)	150	Stanford University(USA)	152
Harvard University(USA)	150	Laval University(Canada)	148
University of Oxford(UK)	150	University of Oxford(USA)	138
University of Pittsburgh(USA)	112	University of Pittsburgh(USA)	125
University of freiburg(Germany)	94	University of Washington (USA)	108
Duke University(USA)	94	Duke University(USA)	106

The University of California, established over 150 years ago, comprises a comprehensive academic and research system featuring 10 world-renowned campuses, 6 academic health centers, 21 health professional schools, and 3 U.S. Department of Energy national laboratories. The UC system serves more than 300, 000 students and employs approximately 266, 800 faculty and staff members. Notably, 8 of its ten campuses are ranked among the top 500 institutions globally in the 2025 QS World University Rankings. This scale—combined with its academic excellence, research output, and societal impact—positions the University of California as a leading force across education, science, health, and public service. From the perspective of inter-institutional collaboration networks, the University of California maintains an extensive global partnership portfolio, engaging in sustained research and academic cooperation with leading institutions worldwide—including the Karolinska Institutet (Sweden), the University of Sydney (Australia), The Chinese University of Hong Kong (China), the University of Oxford (United Kingdom), Laval University (Canada), and prominent U.S. universities such as Harvard University and Yale University.

### Co-authorship among countries/regions

**Figure 8:**
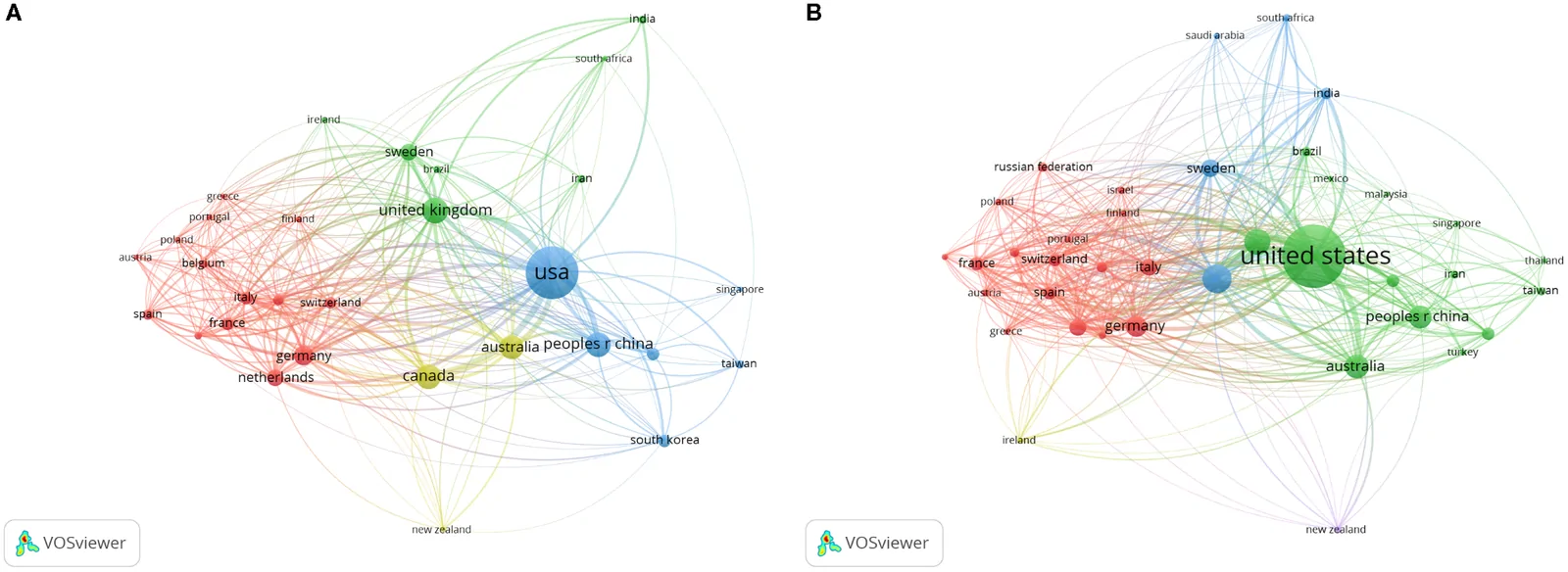
Country/regions distributions of research outputs in CBT-I studies(1996-2025): **(A)** Geospatial collaboration network of countries based on WoSCC. **(B)** Geospatial collaboration network of countries based on Scopus.

The two databases’ analysis of co-authorship at the country- and region-level reveals a consistency in the top three most productive nations—the United States, the United Kingdom, and Canada. Publication outputs and collaborative patterns across other countries and regions are illustrated in **Table 5** and **Figure 8**.

**Table 5:** Top 10 countries/regions with the highest number of publications in CBT-I related research.

WoS		SCOPUS	
Countries/regions	Articles	Countries/regions	Articles
USA	2087	United States	3338
United Kingdom	511	United Kingdom	703
Canada	482	Canada	585
Peoples R China	469	Australia	498
Australia	420	Peoples R China	439
Germany	311	Germany	380
Sweden	224	Sweden	257
Netherlands	203	Netherlands	243
Italy	123	Italy	241
Japan	120	France	161

It is widely acknowledged that the United States currently holds the foremost position globally in terms of comprehensive national power. Its economic and military capabilities, as well as the scope, impact, and quality of its scientific research output, demonstrate this preeminence. According to the volume of publications about CBTI reseach in two databases, the United States leads all countries with over 2000 indexed papers, substantially exceeding the United Kingdom, which ranks second with fewer than one-third that number. Both databases indicate that the United States of America maintains its strongest research ties with Canada and the United Kingdom in terms of international collaboration.

## Terms co-occurrence analysis

**Figure 9:**
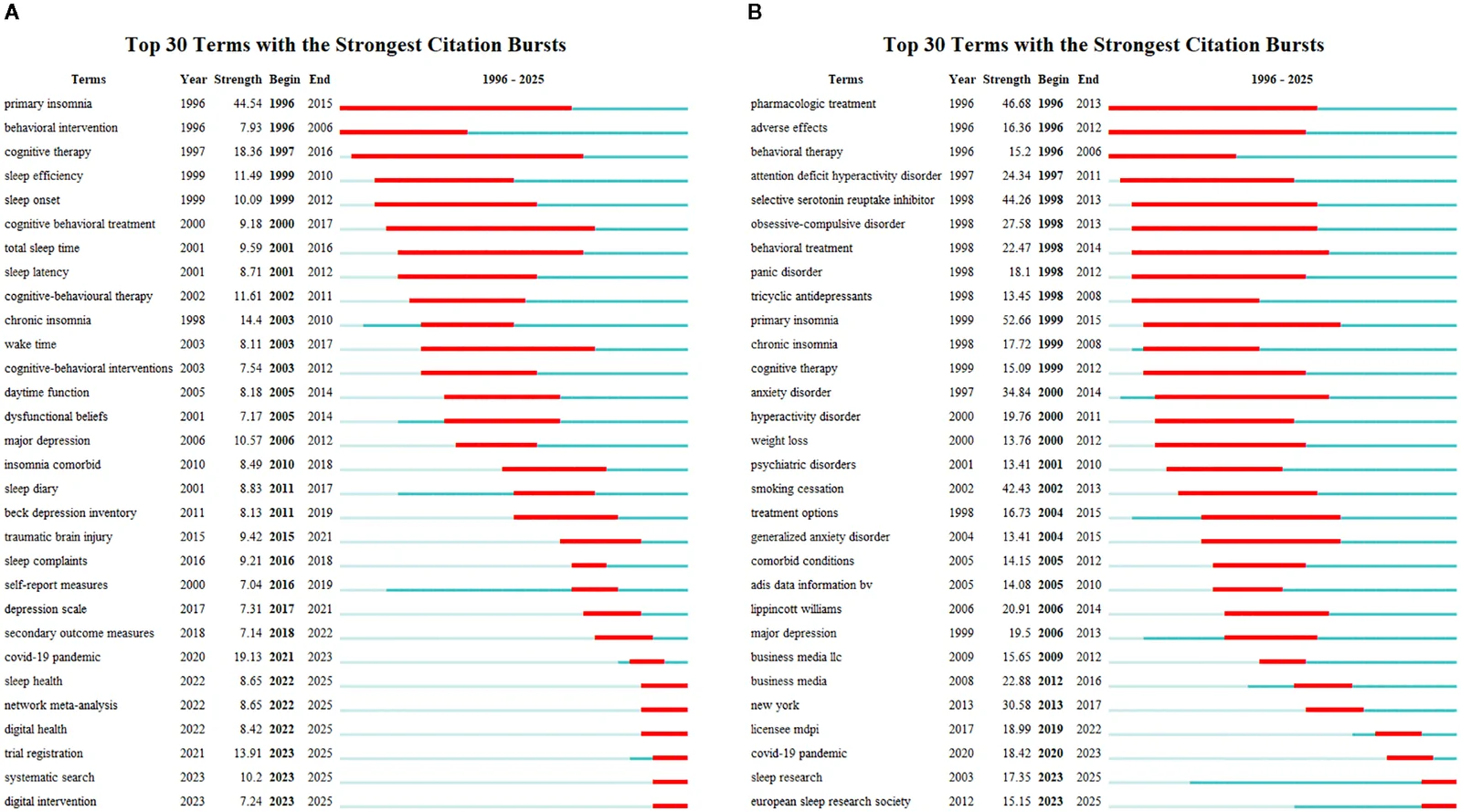
Burstness trends of top 30 terms with the strongest citation bursts in CBT-I related research. **(A)** term burst based on WoS. **(B)** term burst based on Scopus.

Based on a term analysis of the WoS and Scopus databases, this study identifies the top 50 most frequently occurring terms and the top 30 terms exhibiting the strongest citation bursts (**Table 6**; **Figure 9**). A cluster analysis of these terms was also conducted, revealing notable differences in the ranking of high-frequency terms and in the results of burstness analysis.

**Table 6:** Top 50 terms related to CBT-I.

WoS			Scopus		
Count	Year	Term	Count	Year	Term
2171	1996	cognitive behavioral therapy	2806	1997	cognitive behavioral therapy
764	2000	controlled trial	829	1999	controlled trial
685	1996	sleep disturbance	776	1996	sleep disorder
627	2009	insomnia severity index	721	1996	sleep disturbance
609	2002	sleep quality	657	1998	chronic insomnia
501	1997	sleep disorder	588	2004	insomnia severity index
463	1998	chronic insomnia	535	2004	systematic review
446	2011	systematic review	502	1998	sleep quality
435	2003	insomnia symptoms	440	2003	randomized controlled trial
422	2008	primary outcome	431	2008	primary outcome
415	2005	randomized controlled trial	385	2001	clinical trial
370	2004	insomnia severity	371	2002	insomnia symptoms
334	2012	secondary outcome	349	2002	sleep problems
328	1999	sleep problems	346	1996	behavioral therapy
314	2002	control group	346	1998	significant improvement
311	2004	pittsburgh sleep quality index	342	2010	insomnia severity
310	2012	mental health	340	2009	mental health
307	2001	depressive symptoms	324	2000	control group
304	2003	significant improvement	311	2010	insomnia disorder
297	2009	insomnia disorder	291	2012	secondary outcomes
288	1998	clinical trial	276	2000	total sleep time
277	1999	sleep efficiency	274	1996	effective treatment
262	1999	sleep onset	272	1998	future research
261	2001	total sleep time	272	2000	posttraumatic stress disorder
245	2005	posttraumatic stress disorder	271	1996	pharmacologic treatment
239	2001	sleep diary	263	1997	anxiety disorder
221	2004	significant difference	258	1999	sleep efficiency
220	2001	mean age	256	1999	sleep onset
213	1996	behavioral therapy	247	2001	sleep diary
213	2003	future research	242	2003	depressive symptoms
189	1997	sleep hygiene	239	2004	pittsburgh sleep quality index
182	2005	insomnia treatment	234	1998	significant difference
176	2001	dysfunctional beliefs	217	2001	mean age
170	2005	comorbid insomnia	217	2001	primary care
161	2003	effective treatment	206	1999	sleep hygiene
160	2000	chronic pain	205	1999	clinical practice
159	2007	effect size	204	2003	sleep medicine
159	2003	general population	202	1999	major depression
149	2007	sleep onset latency	199	2003	general population
148	2003	primary care	193	2005	comorbid insomnia
147	2006	treating insomnia	187	1999	american academy
143	2010	confidence interval	186	2005	first-line treatment
142	2006	major depression	174	2010	trial registration
134	1997	cognitive therapy	172	2000	risk factor
133	2008	intervention group	171	1999	cognitive therapy
133	1999	sleep restriction	171	2004	obstructive sleep apnea
131	2008	risk factor	168	2001	dysfunctional beliefs
130	2002	anxiety disorder	167	1996	adverse effects
129	1996	primary insomnia	167	1999	behavioral intervention
129	2004	poor sleep	161	2004	chronic pain

In the WoS database, the most frequent term is “cognitive behavioral therapy”, followed by “controlled trial”, “sleep disturbance” and “insomnia severity index” (**Table 6**). The terms with the strongest citation bursts in most recent years (2021-2025) was “covid-19 pandemic”, “sleep health”, “network meta-analysis”, “digital health”, “trial registration”, “systematic search” and “digital intervention” (**Figure 9A**).

**Figure 10:**
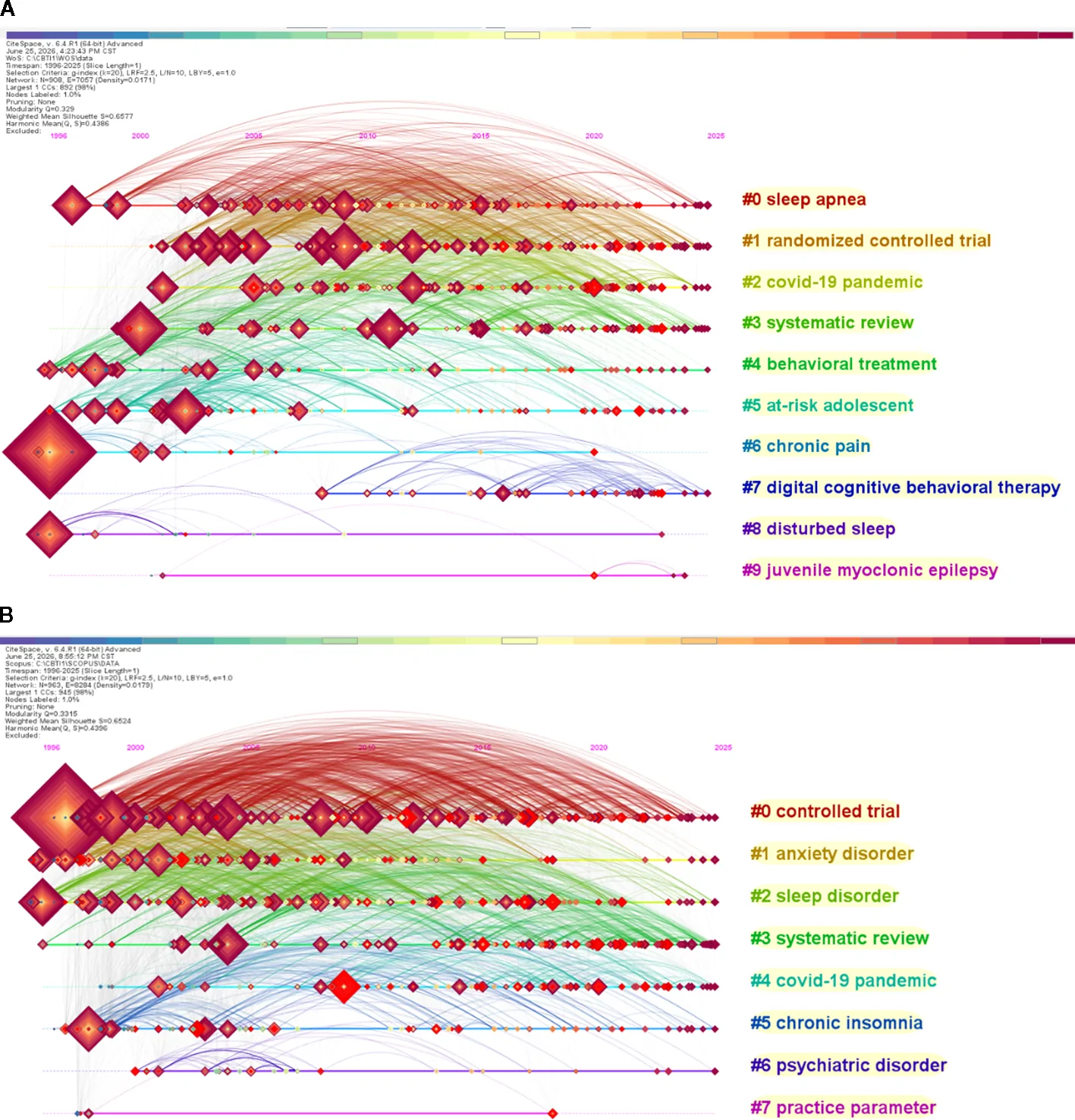
Cluster analysis of terms in CBTI related research. **(A)** term cluster based on WoS. **(B)** term cluster based on Scopus.

Cluster analysis indicates that the terms in the WoS database can be grouped into 10 thematic clusters (**Table 7**; **Figure 10A**). Among the identified clusters, #0, #1, #2, #3, #4, #5, #7, and #9 have consistently represented active research hotspots over the past five years. Term clustering label interpretation adheres to the same principle as the interpretive approach to clustering labels in cited reference analysis.

**Table 7:** Summury of cluster in terms in CBTI(WoS).

ID	Size	Silhouette	Mean (year)	Top 5 terms
0	163	0.61	2012	sleep apnea (9038.77); narrative review (4931.57); parkinsons disease (4469.36); obstructive sleep apnea (3785.43); comorbid sleep disorder (3283.02)
1	155	0.609	2015	randomized controlled trial (5948.95); controlled trial (3596.47); young adult cancer survivor (2134.24); randomised controlled trial (2037.33); large scale (1998.22)
2	138	0.558	2017	covid-19 pandemic (11137.97); mental health (4641.15); posttraumatic stress disorder (3767.85); emotional symptom (3422.46); network analysis (3210.39)
3	109	0.526	2016	systematic review (34859.84); network meta-analysis (8813.62); non-pharmacological intervention (5918.55); nonpharmacological intervention (4050.93); nonpharmacological sleep intervention (2413.28)
4	104	0.745	2006	behavioral treatment (5547.5); american academy (5513.78); sleep medicine (4151.49); chronic insomnia (4084.09); practice parameter (2676.55)
5	94	0.736	2008	at-risk adolescent (3177.57); intensive sleep (2785.98); behavioral therapy intervention trial (2766.34); covid-19 pandemic (2640.01); insomniac complaint (2481.6)
6	47	0.898	2001	chronic pain (8383.83); systematic review (5607.88); cognitive behavioral therapy (4907.34); sleep quality (4637.53); cognitive-behavioral therapy (4002)
7	45	0.791	2019	digital cognitive behavioral therapy (3331.7); cognitive function (3093.18); digital cognitive behavioural therapy (2513.39); real-world evidence (1980.78); empowerment-based cognitive behavioural therapy (1767.2)
8	28	0.91	2001	disturbed sleep (5334.67); sleep disturbance (5091.19); cognitive behavioral therapy (3480.25); effective management (3302.72); disordered sleep (2107.58)
9	9	0.955	2015	juvenile myoclonic epilepsy (176.87); adjunctive behavioural treatment (176.87); clinical characteristics (159.01); naturalistic setting (159.01); body-focused repetitive behavior disorder (159.01)

Accordingly, the existing literature on CBT-I indexed in the WoS database can be systematically organized into the following 8 thematic domains: (a) Expansion of CBT-I applications to comorbid conditions, including obstructive sleep apnea (OSA); (b) Evaluation of CBT-I efficacy in improving sleep quality, as evidenced by randomized controlled trials (RCTs) implementing standardized CBT-I protocols; (c) Context-adapted implementation of CBT-I during the COVID-19 pandemic; (d) Systematic reviews assessing and comparing the relative effectiveness of CBT-I versus other non-pharmacological interventions for insomnia; (e) Policy- and guideline-oriented research led by authoritative bodies—including the American Academy of Sleep Medicine (AASM)—focused on evidence-based behavioral interventions for chronic insomnia disorder; (f) Population-specific adaptations of CBT-I, notably for at-risk adolescents; (g) Emerging research on digital delivery modalities for CBT-I; (h) Intervention strategies targeting sleep disturbances in pediatric populations diagnosed with neurological or neurodevelopmental disorders—such as juvenile myoclonic epilepsy and body-focused repetitive behavior disorder.

In the Scopus database, the most frequent term is “cognitive behavioral therapy”, followed by “controlled trial”, “sleep disorder” and “sleep disturbance” (**Table 6**). The terms with the strongest citation bursts in most 5 years was “covid-19 pandemic”, “sleep research” and “European sleep research society” (**Figure 9B**).

Cluster analysis indicates that the terms in the Scopus database can be grouped into 8 thematic clusters (**Table 8**; **Figure 10B**). Among the identified research clusters, Clusters #0, #1, #2, #3, #4, #5 and #6 have consistently reflected active and evolving research hotspots in CBT-I over the past 5 years.These clusters coalesce around seven thematic foci: (a) Advancing evidence-based implementation of CBT-I through rigorous randomized controlled trials; (b) Evaluating the efficacy and adaptability of CBT-I for anxiety-related conditions—including anxiety disorders, panic disorder, and obsessive-compulsive disorder; (c) Assessing the efficacy and applicability of CBT-I across a spectrum of sleep disorders; (d) Conducting systematic reviews to synthesize evidence on the efficacy and applicability of CBT-I; (e) Context-sensitive adaptations of CBT-I during the COVID-19 pandemic; (f) Policy- and guideline-oriented research led by the AASM for chronic insomnia disorder; and (g) Examining the efficacy and adaptability of CBT-I in comorbid psychiatric disorders.

**Table 8:** Summury of cluster in terms in CBTI(Scopus).

ID	Size	Silhouette	Mean (year)	Top 5 terms (log-likelihood ratio)
0	192	0.704	2011	controlled trial (41899.78); cognitive behavioral therapy (26035.16); randomized controlled trial (18807.78); sleep disorder (14495.38); insomnia symptom (11264.29)
1	190	0.621	2005	anxiety disorder (34181.81); panic disorder (18945.52); obsessive-compulsive disorder (16556.26); smoking cessation (15787.63); pharmacological treatment (15384.14)
2	179	0.597	2008	sleep disorder (63481.14); sleep disturbance (29131.97); narrative review (18884.25); controlled trial (16920.75); common sleep disorder (13085.5)
3	139	0.606	2018	systematic review (58881.81); network meta-analysis (13392.83); scoping review (7877.72); comparative efficacy (5959.49); non-pharmacological intervention (5708.93)
4	101	0.597	2017	covid-19 pandemic (7816.51); sleep disorder (5274.78); healthcare worker (3806.39); mental health (3669.08); app-based intervention (3432.97)
5	98	0.706	2008	chronic insomnia (21734.84); american academy (11022.66); behavioral treatment (9886); systematic review (8659.56); sleep restriction therapy (7103.83)
6	34	0.919	2008	psychiatric disorder (1501.48); bariatric surgery (1278.75); long-term follow-up (1267.86); veteran population (1159.35); economic consequence (920.87)
7	12	0.972	1999	practice parameter (327.39); cancer caregiver (296.52); obsessive- compulsive disorder (280.19); internet-based psychological intervention (263.64); various conventional therapy (247.09)

Due to differences in the scope and composition of the literature indexed by each database, the term analysis results from the WoS and Scopus databases differ significantly. Non-English publications are largely excluded from WoS, whereas the CBT-I-related literature retrieved from Scopus includes multiple languages for this study. Additionally, there is a significant gap between the two databases in the number of CBT-I-related articles indexed—approximately 2000 entries. Therefore, this study incorporates topic-word analysis from the WoS and Scopus databases to thoroughly identify research hotspots in the field of CBT-I. In particular, three complementing metrics are used to identify hotspots: (a) the top ten topic words; (b) terms with the strongest citation bursts (2021–2025); and (c) term clusters that have emerged in the last five years. To ensure conceptual precision and thematic relevance, terms representing lexical synonyms of searching terms (e.g., “cognitive behavioral therapy”, “sleep disturbance”, “sleep disorder”, “chronic insomnia”, “insomnia symptoms”, “sleep quality”), methodological descriptors (e.g., “controlled trial”, “insomnia severity index”, “systematic review”, “randomized controlled trial”, “primary outcome”, “trial registration”) and the official name—or noun phrases extracted from this name—of the organization devoted to sleep research (e.g., “European Sleep Research Society”, “American academy”, “sleep research”) were systematically excluded from the hotspot analysis.

Lastly, over the previous five years, five topic clusters have maintained a high level of research intensity: (a) Adding CBT-I applications to OSA; (b) Using CBT-I in a context-adapted manner during the COVID-19 epidemic; (c) Population-specific modifications of CBT-I for teenagers who are at risk; (d) New studies on CBT-I’s digital delivery methods; (e) Analyzing CBT-I’s effectiveness and adaptability in comorbid psychiatric diseases (especially anxiety-related conditions).

## Discussion

When a scholar dedicates their research to a specific field of knowledge, it is essential to comprehend the underlying structure of that field. This understanding enables effective utilization of existing resources and supports the achievement of research objectives. Moreover, a deep structural analysis allows scholars to identify gaps or deficiencies within the field. By addressing these limitations, the continuous development and refinement of the knowledge domain can be facilitated, ultimately contributing to the formation of a more comprehensive and coherent knowledge system. Therefore, the significance of this study lies in analyzing the intellectual base, research front and research hotspot of the CBT-I field through a bibliometric analysis. This approach aims to enhance our understanding of the field’s historical development and provide valuable insights for its future progression.

### Intellectual base

The intellectual base is constructed from a collection of co-cited literature, which can be identified through the analysis of cited references. Based on the topics covered, the 50 cited articles can be categorized into 8 groups: (a) management guidelines and reference books on insomnia; (b) face-to-face CBT-I in individuals with insomnia disorder; (c) self-help or digital CBT-I interventions for insomnia; (d) CBT-I interventions in mental disorders comorbid insomnia; (e) CBT-I interventions in insomnia comorbid with medical disorders; (f) The overview of information about insomnia; (g) sleep assessment tools; and (h) other relevant topics.

The insomnia management guidelines and reference books serve as a valuable tool for clinical practitioners and an essential educational resource for students and healthcare professionals. These guidelines grade evidence and recommendations using the ACP grading system, which is based on the Grading of Recommendations Assessment, Development and Evaluation (GRADE) approach. This work synthesizes previous research and provides a comprehensive reference framework for future researchers. By building upon these recommendations, insomnia researchers can advance their work more effectively, making these studies indispensable in the field of insomnia research.

The empirical trials of face-to-face CBT-I represent an intervention strategy originally developed for the treatment of chronic insomnia. Through extensive research and in-depth investigation, CBTI has been demonstrated to be an effective intervention for insomnia, thereby providing a valuable reference for the development of targeted strategies to address insomnia symptoms arising from various physiological and psychological factors.

The development and popularization of the internet have provided a new direction for the development of CBT-I, gradually propelling it towards digitalization. Despite its proven efficacy through numerous randomized controlled trials, traditional CBT-I faces limitations in availability and accessibility. Consequently, internet-delivered CBT-I (eCBT-I) has emerged as a timely solution. Studies have shown that eCBT-I yields comparable outcomes to face-to-face CBT-I in improving insomnia, providing robust data support for its widespread implementation. These findings mark a significant advancement in the digitalization of CBT-I, enabling it to better meet diverse patient needs.

Many other mental disorders are associated with insomnia, such as anxiety, depression and PTSD. Therefore, addressing insomnia can aid in the treatment of mental disorders. Comorbid depression and insomnia constitute one of the most prevalent clinical presentations. Early studies primarily emphasized the alleviation of depressive symptoms. Over time, however, the significance of addressing insomnia symptoms has gained increasing recognition. As a result, insomnia-specific interventions have been integrated into the treatment protocols for individuals with depression, contributing to improved outcomes in depressive symptomatology. Given that CBT-I is the first-line psychological intervention for insomnia, its application has progressively expanded to include patients with comorbid mental disorders.

Additionally, certain physical illnesses, such as cancer and chronic pain, can cause insomnia. Effective treatment requires addressing both the underlying disease and insomnia itself. Consequently, the indications for CBT-I have been expanded to include a broader range of insomnia presentations, such as insomnia related to medical conditions. These studies highlight the versatility of CBT-I, allowing therapists and patients to tailor treatment approaches according to specific needs, thereby significantly promoting the adoption of this therapy.

A concise but comprehensive summary of insomnia-related knowledge—including core concepts, epidemiological characteristics, diagnostic and differential diagnostic criteria, and evidence-based treatment options—provides clinicians and researchers with a practical and efficient reference when time is at a premium. Although this content is not directly focused on the practice of CBT-I, it plays a pivotal role in informing clinical practice and guiding future advancements in the field, thus forming an essential foundation for the continued development and implementation of CBT-I.

Whether administered in a face-to-face format or through digital platforms, CBT-I requires the use of appropriate assessment tools in practice to evaluate its effectiveness in alleviating participants’ insomnia. Two widely recognized tools used for this purpose are the ISI and the Consensus Sleep Diary. ISI is a self-rating instrument specifically designed to evaluate the severity of insomnia, aiding researchers in understanding patients’ subjective experiences. ISI has become an important reference in CBT-I research, enhancing the evaluation methods for CBT-I. The adoption of Consensus Sleep Diary for insomnia facilitated comparisons across studies and advance the field.

Other relevant topics included a critical evaluation of CBT-I and the differentiation of pharmacological interventions for the acute and long-term management of insomnia. During the development of CBT-I, some scholars have also reflected on and summarized the deficiencies and challenges that emerged in this process, providing references for establishing a more complete CBT-I system. Key issues include a shortage of CBT-I providers, insufficient service capacity due to lack of knowledge and motivation among providers, and limited patient awareness and willingness to engage in CBT-I. Addressing these challenges systematically can better promote the development and application of CBT-I. In addition, despite the fact that pharmacological treatments for insomnia are not central to CBT-I, having a clear understanding of how they differ from CBT-I is clinically necessary for its successful implementation. Providers of CBT-I should be well-versed in hypnotic medication use guidelines based on evidence. This knowledge aids in the correction of patients’ misconceptions regarding pharmacological treatment, improves compliance with clinical recommendations, and reduces the dangers of irregular or inappropriate medication use—factors that could otherwise jeopardize the efficacy of CBT-I and prevent long-term improvements in sleep.

Clarifying the intellectual base can assist young researchers who are new to the field of scientific research in understanding the foundational knowledge structure of their discipline, establishing a comprehensive grasp of its developmental trajectory, and laying a solid foundation for undertaking novel research.

### Research front

The research front is delineated by literature that cites these intellectual base, which can be inferred from the clustering analysis of these citations. Therefore, the major research frontiers in the field of CBT-I over the past 5 years include: (a) emphasizing on the application of digital CBT-I models; (b)investigating CBT-I interventions for insomnia in patients with OSA; and (c) exploring the application of CBTI for insomnia among cancer patients.

Digitalized CBTI is distinguished by its enhanced accessibility, cost-efficiency, and scalability—attributes that have significantly advanced its implementation, dissemination, and scholarly attention as a leading area of research. Historically, CBT-I delivery relied predominantly on face-to-face interventions, which facilitated real-time observation of participant responses and immediate behavioral feedback, thereby supporting robust therapeutic fidelity and clinical effectiveness. However, this modality posed notable constraints: limited intervention capacity (typically fewer than 20 participants per session) and variability in procedural standardization due to inter-clinician differences—factors that collectively impeded the broader adoption of CBT-I in clinical and public health settings for insomnia management. The advent of digitalized CBT-I has effectively addressed these limitations. In this paradigm, evidence-based CBT-I protocols are systematically operationalized into standardized, software-delivered interventions, ensuring consistent content delivery across all participants. This uniformity mitigates clinician-dependent variability in treatment fidelity and enhances reproducibility—particularly critical when scaling interventions across diverse populations. Furthermore, digital delivery eliminates logistical barriers such as travel time and scheduling conflicts, thereby improving participant engagement, adherence, and recruitment feasibility.

Since about 2015, research on digital CBT-I has been a major focus, and it is still an active field of study today. The parallel development of fourth-generation (4G) mobile communication infrastructure, which has enabled wider access to and delivery of digital health interventions, is probably the reason for this ongoing interest. An important development in mobile broadband infrastructure occurred in 2014 with the worldwide deployment of 4G mobile communication networks. This technological change created a strong technical basis for the digital delivery of CBT-I, significantly increased mobile internet speeds, and allowed interactive applications to run smoothly. As a result, CBT-I started moving toward digitally mediated, scalable application, greatly increasing its accessibility. This trajectory was further reinforced in 2019 by the commercial deployment of fifth-generation (5G) networks, which provided ultra-low latency, increased bandwidth, and improved stability. This strengthened the viability and fidelity of real-time, multimedia-rich digital CBT-I therapies. The COVID-19 epidemic that followed, which was highly transmissible and required extensive physical distance, sped up the implementation of remote behavioral health solutions and sparked a wider integration of digital CBT-I into clinical and public health practice.

Similarly, research in oncology has revealed a high prevalence of insomnia among cancer patients. For individuals diagnosed with cancer—particularly those in the intermediate and advanced stages—the condition often represents a profound existential threat, prompting significant psychological distress and forcing patients to confront mortality. This emotional burden frequently contributes to the development of anxiety and depression, which are well-documented precipitants of sleep disturbances. Furthermore, in advanced-stage cancer patients, persistent disease-related pain imposes substantial physical discomfort, significantly impairing the ability to achieve restful sleep. Consequently, scholars began investigating the potential of CBT-I as an intervention for cancer patients, targeting sleep disruptions caused by both psychological distress and physical symptoms. However, there is currently conflicting empirical data regarding the effectiveness of cognitive behavioral therapy for insomnia (CBT-I) in reducing sleep disturbances in cancer patients. A recent Cochrane systematic review found that CBT-I has only minor effects in this population (61), despite the fact that multiple studies show clinically significant improvements in sleep outcomes after CBT-I intervention (62).

Early research on the relationship between CBT-I and cancer was conducted; the first pertinent publication in the WoS database dates to 2001 (63). Less than ten papers were published annually in this subject before to 2014, suggesting that CBTI-cancer research just started to become a distinct and active field of study after 2014. Dr. Josée Savard of the Cancer Research Centre at Laval University submitted the most peer-reviewed articles, many of which were funded by the Canadian Breast Cancer Research Alliance. Other significant cancer-related organizations, such as the Canadian Cancer Society, the Alberta Cancer Board, and the National Cancer Institute (NCI) of the National Institutes of Health (NIH) in the United States, provided money to more researchers. The advancement and consolidation of CBTI-cancer research as a strong and expanding field of clinical and translational inquiry has been greatly aided by these institutions’ consistent financial support.

Although patients with sleep apnea do not typically exhibit overt symptoms of insomnia, their overall sleep quality is significantly compromised, and their sleep architecture is predominantly characterized by prolonged periods of light sleep. During the daytime, these patients often experience impaired cognitive and physical functioning, manifesting in symptoms that closely resemble those of insomnia. Many individuals misinterpret these symptoms as primary insomnia, leading to misconceptions about sleep and the development of maladaptive sleep behaviors. These incorrect beliefs and habits further exacerbate existing sleep disturbances. In light of these findings, an increasing number of researchers have sought to incorporate CBT-I into the treatment protocols for OSA, particularly in conjunction with non-invasive positive pressure ventilation, aiming to address dysfunctional sleep-related cognitions and behaviors. As a result, CBT-I interventions for OSA patients emerged as a prominent area of academic interest.

Although there has been intellectual interest in the connection between CBT-I and OSA management since 2004 (64), there wasn’t much empirical study done on the subject until about 2015. In its 2016 clinical practice recommendation for the treatment of adult insomnia, the American College of Physicians (ACP) specifically recommended CBT-I as the first-line treatment for chronic insomnia. The potential benefit of CBT-I in comorbid diseases with symptoms of insomnia has since drawn further attention from academics. Notably, new research backs the use of CBT-I in the multidisciplinary treatment of OSA, where it targets maladaptive sleep-related thoughts and behaviors to supplement continuous positive airway pressure (CPAP) therapy (65). As a result, since 2016, the relationship between CBT-I and OSA has drawn increasing clinical and scientific interest.

The most recent developments and development trends are represented by the research front. Analyzing the research frontiers within the CBT-I field enables researchers to better understand current research directions and avoid duplication of existing work. In addition, researchers can improve the quality and impact of their own research by adopting methods and techniques from cutting-edge studies. Additionally, this kind of analysis provides fresh viewpoints and insights, encouraging academics to investigate novel directions and emerging research fields.

### Research hotspot

The “Research Base” and “Research Front” sections of CBT-I already cover topics like the CBT-I applications to OSA, digital delivery modalities for CBT-I, the efficacy and adaptability of CBT-I in comorbid psychiatric disorders (including anxiety-related conditions). The “Research Hotspot” section does not return to these subjects to avoid repetition. This section, on the other hand, focuses on how CBT-I was adapted and implemented during the COVID-19 pandemic, the population-specific adaptations of CBT-I for at-risk adolescents.

Five years ago, the COVID-19 virus spread globally. Its characteristics—strong infectivity, high fatality rate, and the absence of specific treatment options—caused widespread public anxiety and triggered mental health issues such as anxiety disorders and insomnia. During this unprecedented period, the application of CBT-I to address sleep disturbances became critically important for preserving psychological well-being and improving quality of life. Consequently, sleep disorders associated with the impact of COVID-19 have emerged as a key research focus over the past five years and have become a prominent topic in contemporary academic inquiry. Meanwhile, due to the contagious nature of the virus, face-to-face delivery of CBT-I has been limited, thereby accelerating the development and adoption of digital CBT-I platforms. Digital CBT-I has emerged as a promising trend, offering more accessible and efficient therapeutic options for individuals suffering from insomnia. As a result, researchers worldwide have conducted localized empirical studies, contributing to the growing body of literature on digital CBT-I and establishing it as a significant research frontier in recent years.

Insomnia among adolescents is frequently comorbid with underlying mental and psychological disorders, which often serve as primary contributors to sleep disturbances. Consequently, conventional clinical management typically prioritizes the identification and treatment of these root conditions. In recent years, however, the application of CBT-I has expanded beyond adult populations, with increasing empirical support for its adaptation to adolescent cohorts (66, 67). When integrated into standard care—either as a standalone intervention or as an adjunct to treatment for primary psychiatric conditions—CBT-I not only improves sleep outcomes but also enhances overall therapeutic efficacy for associated mental health concerns. Due to their upbringing in a digitally immersive environment, Generation Z adolescents have unique cognitive and behavioral characteristics, such as digital literacy, a strong inclination toward peer-mediated and socially embedded experiences, and a preference for personalized and interactive modalities. These characteristics render them particularly receptive to digitally delivered CBT-I, thereby improving intervention accessibility, engagement, and scalability. Research on CBT-I implementation for at-risk adolescents has become a major focus in the literature on pediatric sleep and mental health due to the convergence of clinical need, empirical promise, and developmental appropriateness.

### Limitations

The big data capabilities of CiteSpace and VOSviewer software can significantly streamline the process of evaluating literature research content by directly extracting relevant information, thereby facilitating the screening of literature for analysis. However, this approach may also lead to an over-reliance on a single evaluation metric and the subjectivity in software parameter settings, which could potentially compromise the credibility of the findings. Given that the primary focus of this study is on the references cited in papers and keywords within the CBT-I field, it is not feasible to conduct in-depth evaluations of the content and quality of each individual reference. Although this study leveraged the WoS to ensure high-quality, rigorously indexed literature, and employed the Scopus database to broaden coverage across disciplines and publication types, the scope of included literature remains inherently constrained by database selection criteria. Consequently, the analysis cannot claim comprehensive representation of all extant research on CBTI. Furthermore, the cited reference analysis relied exclusively on the WoS Core Collection, limiting cross-database comparability and potentially introducing bibliometric bias in citation-based findings. These limitations may, to some extent, affect the overall comprehensiveness and scholarly value of this study.

## Conclusion

Through co-citation analysis of cited references, keyword co-occurrence analysis, and co-authorship analysis across authors, institutions, and countries/regions, this study mapped the intellectual landscape of CBT-I. Management guidelines and reference books on insomnia, face-to-face CBT-I interventions for people with insomnia disorder, self-help or digital CBT-I interventions for insomnia, CBT-I applications in mental disorders comorbid insomnia, CBT-I interventions for insomnia comorbid with other medical conditions, The overview of information about insomnia, sleep assessment tools, and other related topics were the eight primary knowledge bases that were found. The application of digital CBT-I models, CBT-I interventions for cancer and management of insomnia associated with sleep apnea are three emerging research frontiers in the CBT-I domain over the past 5 years. Morin CM is the most prominent researcher in the field in terms of scholarly contributions. University of California has emerged as the leading institution in CBT-I research. United States produces the most CBT-I-related research in the world. Adding CBT-I applications to OSA, using CBT-I in a context-adapted manner during the COVID-19 epidemic, population-specific modifications of CBT-I for teenagers who are at risk, new studies on CBT-I’s digital delivery methods, assessing CBT-I’s effectiveness and adaptation in comorbid psychiatric diseases (especially anxiety-related conditions) are all current research hotspots in CBT-I.

## Data Availability

The original contributions presented in the study are included in the article/supplementary material. Further inquiries can be directed to the corresponding author.
